# Prevalence of Metabolic Syndrome and Its Associated Risk Factors Among Schoolchildren Aged 11–13 Years Living in Thiruvananthapuram District, Kerala, India: A Nested Case-Control Study

**DOI:** 10.7759/cureus.72994

**Published:** 2024-11-04

**Authors:** Sreeja S Aravindakshan, Anita David, Geetha Saradakutty, Prakash Agarwal

**Affiliations:** 1 Department of Child Health Nursing, Government College of Nursing, Thiruvananthapuram, Thiruvananthapuram, IND; 2 Department of Paediatric Nursing, Sri Ramachandra Institute of Higher Education and Research, Chennai, IND; 3 Department of Paediatrics, Government Medical College, Thiruvananthapuram, Thiruvananthapuram, IND; 4 Department of Paediatric Surgery, Sri Ramachandra Institute of Higher Education and Research, Chennai, IND

**Keywords:** adolescent diabetes, associated factors with paediatric metabolic syndrome, metabolic syndrome, physical activity, school children, screentime

## Abstract

Introduction: Metabolic syndrome is a cluster of conditions that includes increased blood pressure, increased blood sugar, excess body fat around the waist, and a rise in triglyceride levels which could increase the risk of developing type 2 diabetes and cardiovascular diseases. While metabolic syndrome is typically associated with adults, concern is growing about its prevalence and implications among children and adolescents. The rapid rise in childhood obesity and physical inactivity has led to an increase in risk factors among young populations, making it a pressing public health issue. This study aims to investigate the risk of metabolic syndrome among children aged 11-13 years, exploring its association with various sociodemographic and clinical factors.

Methods: A nested case-control study was conducted from June 2021 to November 2021 among a cohort of school children aged 11-13 years in Thiruvananthapuram district, Kerala, India. Twenty-two clusters (schools) were identified from the schools in Thiruvananthapuram’s educational subdistrict using populations proportionate to size. Data on the sociodemographic characteristics and lifestyle practices of 1,580 schoolchildren and their parents were collected using a questionnaire. Body mass index, waist circumference, and blood pressure were also assessed to screen children for metabolic syndrome. Fasting blood glucose, triglycerides, and high‑density lipoprotein levels were evaluated in 57 cases (children with metabolic syndrome after screening) and 116 controls (children without metabolic syndrome after screening).

Results: The prevalence of pediatric metabolic syndrome as defined by the International Diabetes Federation (IDF) classification in the sample was 3.6% (n=57) (95%CI 1.6-6.6). Modifiable and nonmodifiable risk factors found to be associated with pediatric metabolic syndrome after multivariate analysis were gender, decreased physical activity, especially organized physical activity and moderately vigorous physical activity or play, skipping meals, sleeping for less than eight hours at night, eating with screens on, use of screens for more than two hours daily, and preference for soft drinks or carbonated drinks over water when thirsty. This study also evaluated the predictive power of the associated factors for pediatric metabolic syndrome using receiver operating characteristic (ROC) analysis. The ROC curve showed an area under the curve of 0.926 (95% CI: 0.891-0.961, *p* < 0.001), indicating high predictive power.

Conclusion: The findings of this study brought out evidence of an escalation in the rate of metabolic syndrome among children in their early adolescent stage. This rise is a trend with increasing sedentary time and the overuse of screens by the younger generation in this modern era of gadgets and technologies. Our findings would act as a catalyst in implementing community and school-based activities to improve physical activities and lifestyle modifications among children, thereby reducing the risk of early development of metabolic syndrome.

## Introduction

The increasing physical inactivity leading to a rise in obesity among children and adolescents is a major threat to health in the 21st century. In 2022, 37 million children under the age of five years were overweight, and over 390 million children and adolescents aged 5-19 years were overweight, including 160 million who were living with obesity [1]. Around 200 million schoolchildren are thought to be overweight or obese globally, according to estimates from the International Obesity Task Force and the International Association for the Study of Obesity [2].

Developing countries such as India have an unprecedented “dual hardship,” with obesity among children and adolescents at one end of the continuum and malnutrition and underweight at the other. The rates of overweight and obesity in children and adolescents are rising irrespective of the income status in society, pointing to the urgency of an equitable and conscious strategy for economic and nutritional changes to effectively defend against this dual hardship in India [3]. The prevalence of overweight or obesity among adolescents aged 15-19 years was 4.2%, according to the National Family Health Survey (NFHS-4) 2015-16, a substantial increase from the previous surveys [4]. The survey also found that 5.74-8.82% of schoolchildren in India were obese; in urban South India, 21.4% of boys and 18.5% of girls are either overweight or obese. The detection of metabolic syndrome (MetS) is usually missed in the early stages of development as in the case of children and adolescents. Early recognition and prompt treatment are crucial for better results. Changes in dietary habits and increasing physical activity are the mainstay of management [5]. Children with obesity are more likely to develop diseases like diabetes and cardiovascular diseases at a younger age. Obesity is assumed to be the final product of an increase in caloric and fat intake, even though it is a disorder of multiple causes. The steady decline in physical activity also has been playing an imperative role in the rising prevalence of obesity [6].

MetS is a group of metabolic risk conditions that include disturbance in glucose metabolism, dyslipidemia, abdominal obesity, and arterial hypertension. MetS risk includes obesity plus two or more risk factors as specified by the International Diabetes Federation (IDF) criteria: waist circumference (WC) ≥ 90th percentile, fasting glucose ≥ 100 mg/dL, systolic blood pressure (BP) ≥ 130 mmHg, diastolic BP ≥85 mmHg, triglyceride (TG) ≥150mg/dL, and high-density lipoprotein (HDL) <1.03 mmol/L (<40 mg/dl) [7]. A systematic review of MetS among adolescents in India observed that the pooled prevalence of MetS was 3.4% (95% confidence interval (CI): 1.1-6.6%) using the IDF criteria and 5.0% (95%CI: 3.3-6.9%) using the National Cholesterol Education Program-Adult Treatment Panel III criteria [8]. This prevalence, higher than in other countries, poses a threat to adolescents’ health and demands urgent intervention.

A population-based study conducted in various states of India in 2016-18 showed an increasing prevalence of MetS estimated at 14.2 million adolescents in India having MetS [9]. There was huge variation in the prevalence of MetS among states, ranging from 0.5% to 16.5%. The rate was estimated to be 5.9% in Kerala, with a large difference in other states. The prevalence of MetS was 3.3% among 10-16-year-olds residing in Shimla, Himachal Pradesh. In Andhra Pradesh, South India, a prevalence of 4.0% was found in school-aged adolescents, indicating a high percentage of overweight children with MetS [10]. MetS refers to a cluster of risk factors for coronary artery diseases (CAD) such as insulin resistance, abdominal obesity, impaired glucose, elevated blood pressure, elevated triglycerides, and reduced high-density lipoproteins (HDL) [11]. Many studies globally have reported the prevalence of MetS as ranging from 0.2% to 38.9% among children, and it is more prevalent among the overweight (11.9%) and obese (29.2%) population [12].

The prevalence of MetS among children with obesity was 56%, with a mean age of 11.3 ± 2.73 years. Multiple logistic regression analysis showed age and sedentary lifestyle were the significant factors associated with MetS among obese children. Obese children in the older age range had 1.27 times higher odds of having MetS while those with sedentary lifestyles had 3.57 times higher odds of having MetS than those who were non-sedentary [12]. Overweight and obesity are important features, along with changes in glucose metabolism, dyslipidemia, and hypertension. Disorders associated with MetS include fatty liver, polycystic ovarian syndrome (PCOS), and pro-inflammatory states [13]. Children who frequently interrupt their sedentary time may experience lower levels of cardiometabolic risk than those who accumulate sedentary behaviour with less frequent interruption. There is a need to increase physical activity among children, and to educate parents and children on lifestyle modification [14].

Studies on the prevalence of MetS among children in Kerala are limited, especially among preadolescents in the age group of 11-13 years. Hence, this study aims to determine the prevalence of MetS and its associated risk factors among schoolchildren aged 11-13 years in Thiruvananthapuram, Kerala, India.

## Materials and methods

This was a nested case-control study of schoolchildren conducted in the Thiruvananthapuram district of Kerala, India. The study was approved by the Institutional Ethical Committee of Sri Ramachandra Institute of Higher Education & Research (approval number: IEC-NI/19/NOV/71/86). Permission was also obtained from the Directorate of Public Instructions (Kerala) (approval number: approval number is A4/29166/2019/DGE) and school authorities. In addition to informed consent from parents, assent from children was also obtained.

Study population

A cohort of 1,580 children aged 11-13 years from schools in the Thiruvananthapuram district were screened for MetS between June 2021 and November 2021 according to the IDF definition. The inclusion criteria were: children aged 11-13 years studying in schools of Thiruvananthapuram district, who had consented to participate (and whose parents consented), and who had met the MetS criteria of the IDF definition. Children with any endocrine disorders, mental disorders, or were on steroid or hormonal treatments were excluded from the study.

Sample size calculation

The sample size for the prevalence estimation was calculated from previous studies based on the study objectives and the sample size was fixed to 887 using a single proportion formula. The sample size was calculated based on the prevalence of obesity of 10.4% among children residing in Kerala, precision of 80%, and a standard value (Z) of 1.96) [15]. To justify the clustering effect, the sample size computed was multiplied by 1.5, resulting in 1,580.

The sample size for associated factors was calculated by the statistical software Epi Info (Centers for Disease Control and Prevention, Atlanta, Georgia, United States) using percentage of exposure in controls, odds ratio (OR), type 1 error (Zα) at 95%, and power(1-β) at 80%. Hence, the percentage of exposure to controls and the odds ratio for the risk factors (sedentary lifestyle (adjusted OR 3.57, 95% CI: 1.48 to 8.59) were used to estimate the case and control sample in the ratio of 1:2, Zα at 95% and power(1-β) at 80% [15]. The sample size for cases was taken as the number of children with MetS, i.e. 57, and thus the number of controls was taken as approximately twice that, i.e. 116.

Sampling technique

**Figure 1:**
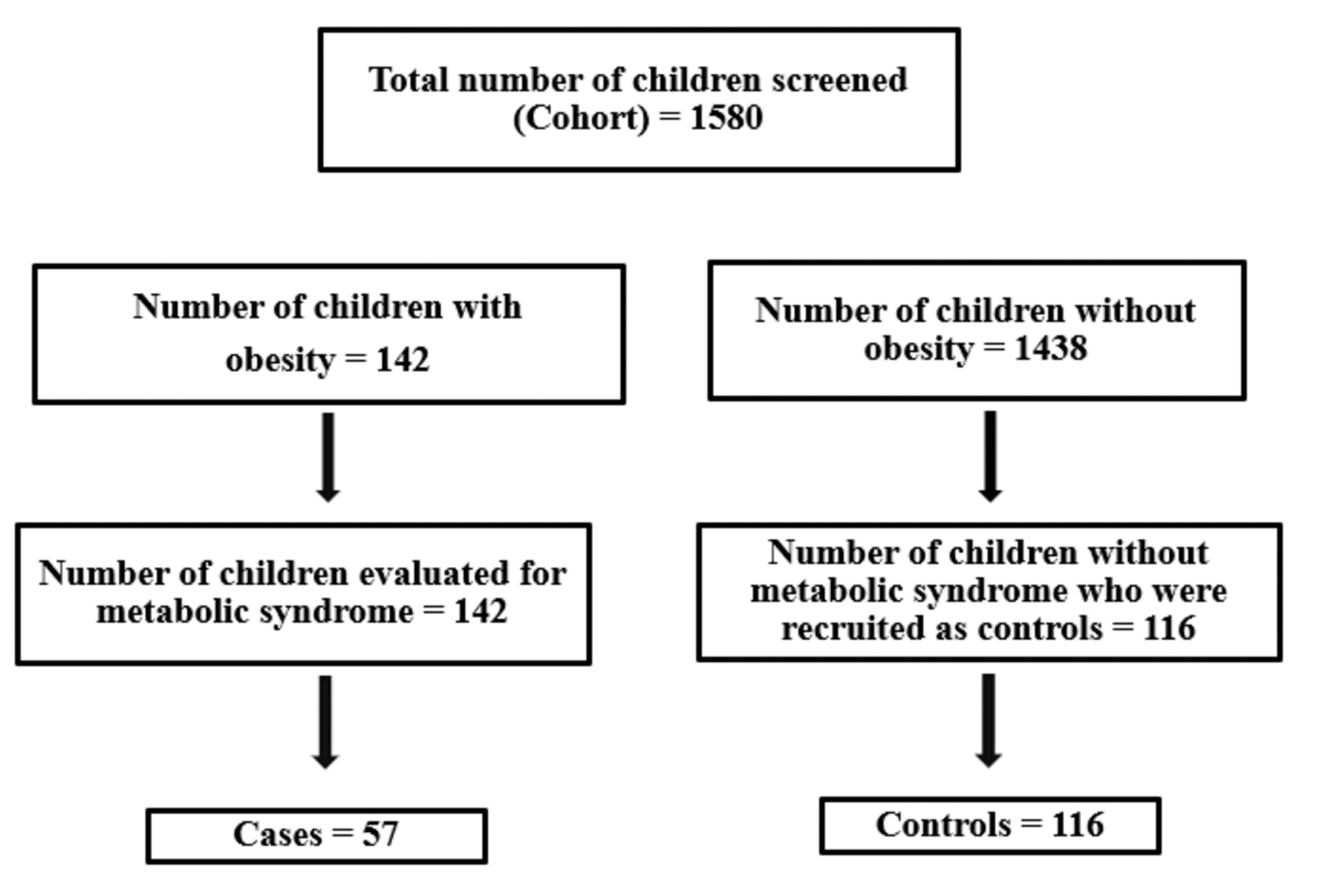
Recruitment of cases and controls

Stratified cluster random sampling was used to select the study participants. According to the categorization of schools in Kerala, schools were stratified as government, private aided, and private unaided. Children were selected proportionately according to the number of students in the sectors. Figure 1 depicts the selection of the study participants.

At first, 22 clusters (schools) were identified from the list of 1,420 schools in the education subdistrict of Thiruvananthapuram. From each school, approximately 70-72 children in the age group of 11-13 years who were studying in grades 6 and 7 were selected. Of the 22 clusters, 10 were government schools, eight were private-aided schools, and four were private unaided schools. The total number of schoolchildren in Thiruvananthapuram district was 295,430. The proportion of students from each stratum was calculated as a percentage of the total number, so the number of children selected from each stratum included 738 students (46.7%) from government schools, 582 students (36.8%) from private aided schools, and 260 students (16.5%) from private unaided schools [16]. Using a simple random technique in each school, one or two classes were selected in which each class had nearly 30-35 students.

Data collection

Data regarding the sociodemographic profile and lifestyle practices of children were obtained from parents through interviews, using a parental questionnaire and lifestyle checklist. All children then underwent anthropometric assessment, and children with obesity were identified by calculating the BMI for each child individually using the Ped(z) application (https://www.pedz.de/en/welcome.html), as the value varies according to gender and date of birth. BP was also assessed in all children. Fasting blood glucose, TG, and HDL levels were evaluated in 57 cases and 116 controls. Cases were defined as children aged 11-13 years having metabolic syndrome based on the IDF criteria. Controls were children aged 11-13 years without metabolic syndrome.

Statistical analysis

Data analysis was performed using IBM SPSS Statistics for Windows, Version 20.0 (Released 2011; IBM Corp., Armonk, New York, United States). The sociodemographic profiles and the proportions of children who met each criterion of metabolic syndrome were compared in cases and controls by Pearson chi-square. The OR of case and control status was determined, and multivariate binary logistic regression was used to control for confounding. Multivariate analysis was carried out on two criteria, i.e. those variables with a p-value of <0.25 in univariate analysis and those variables with literature evidence of biological plausibility as an associated factor. The goodness-of-fit of the regression model was assessed by the Hosmer-Lemeshow test. The predictive power of associated factors for pediatric metabolic syndrome was evaluated using receiver operating characteristic (ROC) analysis.

## Results

Cases and controls were derived from the baseline cohort of 1,580 schoolchildren, and the associated factors were also determined. The regression analysis for predictive power was conducted after testing normality using a Q-Q plot and the Shapiro-Wilk test (W = 0.98 at p-value = 0.23) which showed that the data were normally distributed.

Table 1 shows that 64.4% (n=1018) of the children were in the age group of 12-13 years, of which 745 (47.2%) were boys and 835 (52.8%) were girls. Regarding income status, over three-quarters (n=1227, 77.7%) were above the poverty line. Over two-thirds (n=1119, 70.8%) were from nuclear families. Analysis of birth order showed that 574 (36.3%)were secondborn.

**Table 1:** Sociodemographic variables of participants (N=1580) APL: above poverty line; BPL: below poverty line

Variables		Frequency	Percentage
Age in years	11–12	562	35.6
	12–13	1018	64.4
Gender	Male	745	47.2
	Female	835	52.8
Income	APL	1227	77.7
	BPL	353	22.7
Birth order	First	972	61.5
	Second	574	36.3
	Third	32	2.0
	Fourth or higher	2	0.1
Type of family	Nuclear	1119	70.8
	Extended nuclear	193	12.2
	Joint	268	17.0

Table 2 shows that 208 (13.2% )children were underweight and 142 (9.0%) were obese. Among the 1,580 children, 36 ( 2.3%) had a systolic BP of more than 130 mmHg, and 35 (2.2%) had a diastolic blood pressure of more than 85 mmHg.

**Table 2:** Clinical variables of the participants (N=1580) BMI: body mass index; BP: blood pressure; SD: standard deviation

Variable		Frequency (n= 1580)	Percentage
Nutritional status according to BMI	Normal weight (5^th^- 85^th^ Percentile)	996	63.0
	Overweight (> 85^th^ – 94^th^ Percentile)	234	14.8
	Obesity (>/= 95^th^ Percentile)	142	9.0
	Underweight (<5^th^ Percentile)	208	13.2
Nutritional status according to Z score	Normal weight (+1 SD to +2 SD)	1085	68.7
	Overweight (>+1 to 2SD)	355	22.5
	Obesity (>2 SD)	36	2.3
	Underweight (< -2SD)	104	6.6
Waist circumference	5^th^ to 85^th^ Percentile	991	62.7
	> 85^th^ to 94^th^ Percentile	236	14.9
	>/= 95^th^ Percentile	142	9.0
	<5^th^ Percentile	211	13.4
Systolic BP in mmHg	90-110	1314	83.2
	110-120	197	12.5
	120- 130	36	2.3
	>130	33	2.1
Diastolic BP in mmHg	50-60	556	35.2
	60-70	769	48.7
	70-85	220	13.9
	>85	35	2.2

It is evident from Table 3 that nearly three-quarters (n=1157, 73.2%) of the children used a vehicle to travel to school, over two-thirds (67.6%) used screens for more than two hours daily, and over half (n=864, 54.7%) engaged in an organized physical activity for more than three days in a week. Moreover, 1283 (81.2%) ate three meals daily, 1334 (84.4%) preferred water to other drinks when thirsty, and 1169 (74%) were in the habit of sleeping for more than eight hours at night.

**Table 3:** Distribution of participants’ lifestyle practices PE: physical education; MVPA: moderately vigorous physical activity ^a^ E.g., aerobic exercises, swimming, cycling, dancing for 30 minutes at least three days a week; ^b^ Fried food, chips, biscuits, cakes, sweets, etc. twice or more in a week.

Variable		Frequency	Percentage
Use of bike/walking to school	Yes	423	26.8
	No	1157	73.2
Participation in PE	Yes	1346	85.2
	No	234	14.8
Organized physical activity^a^	Yes	864	54.7
	No	716	45.3
Eats 3 meals daily	Yes	1283	81.2
	No	297	18.8
Eats fruits daily	Yes	873	55.3
	No	707	44.7
Eats vegetables daily	Yes	948	60.0
	No	627	39.7
Eats food from outside twice or more in a week	Yes	804	50.9
	No	776	49.1
Eats junk food^b^	Yes	1235	78.2
	No	345	21.8
Prefers to drink water when thirsty	Yes	1334	84.4
	No	246	15.6
Eats with TV or any other screen on	Yes	1037	65.6
	No	543	34.4
Sleeps for at least 8 hours at night	Yes	1169	74.0
	No	411	26.0
Screen time in a day	Less than 2 hours	512	32.4
	More than 2 hours	1068	67.6
MVPA or play for 60 minutes a day in a week	Less than 3 days	913	57.8
	More than 3 days	667	42.2

Prevalence of MetS

**Figure 2:**
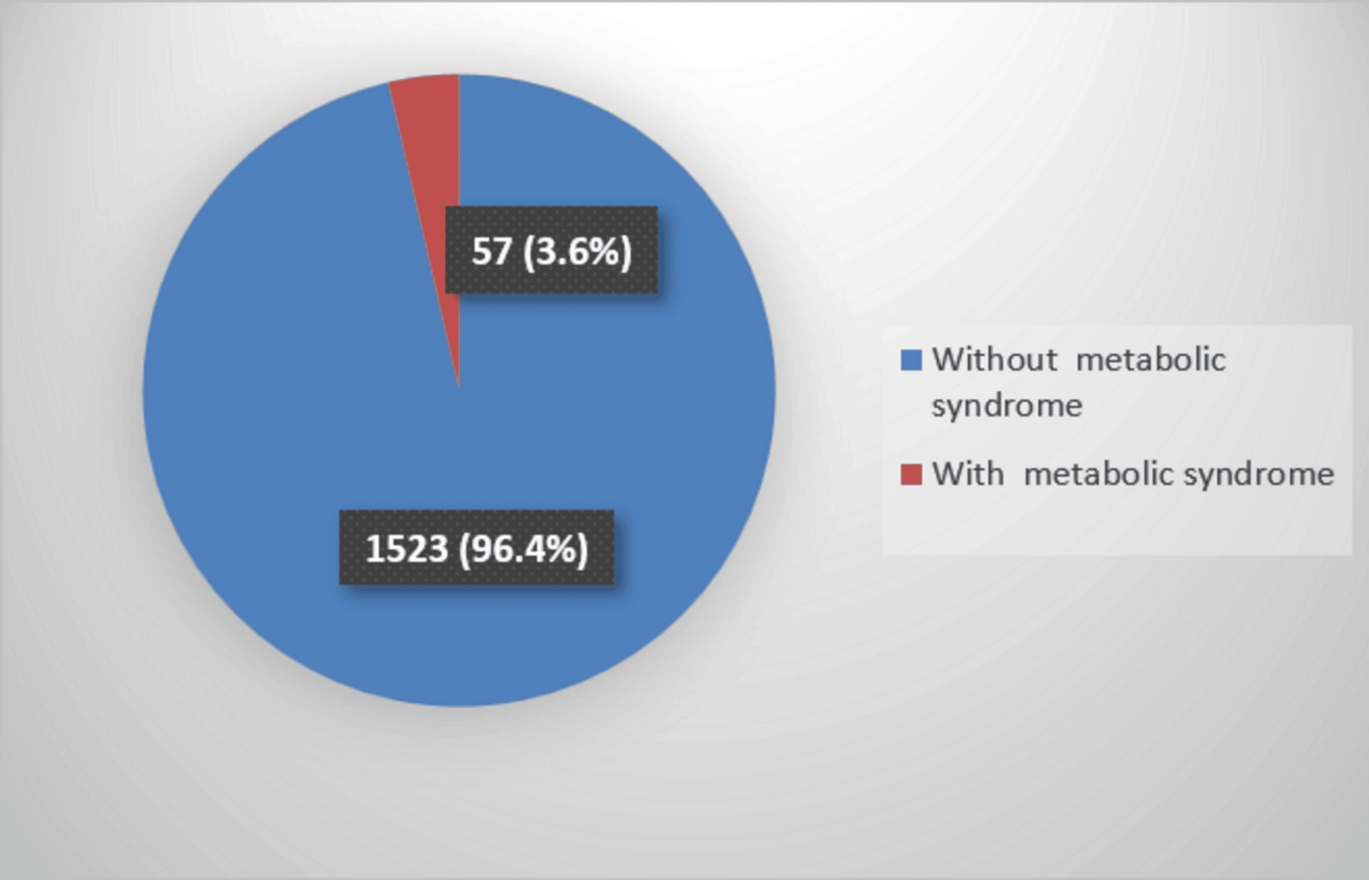
Prevalence of metabolic syndrome

Figure 2 shows that the prevalence of MetS in the sample was 3.6% (n=57) (95%CI: 1.6-6.6). 

Table 4 shows the distribution of components of MetS as per IDF criteria in cases and controls, which shows that systolic BP of more than 130 mmHg was found in 26 (45.6%) cases and only in three (2.6%) controls. Also, it shows that diastolic BP of more than 85 mmHg was present in 16 (28.1%) cases. It is also evident that the fasting blood glucose was more than 100 mg/dl in 31.6% (n=18) of cases and 1.8% (n=2) of controls, HDL was less than 40 mg/dl in 22.8% (n=13) of cases and 0.90% (n=1) of controls whereas TG was more than 150 mg/dl in 15.8% (n=9) of cases.

**Table 4:** Distribution of components of metabolic syndrome as per IDF criteria in cases and controls IDF: International Diabetes Federation

Components of metabolic syndrome		Group		Total	X^2^	P Value
		Cases (n=57), n (%)	Controls (n=116), n (%)			
Systolic BP	< 130 mmHg	31 (54.4 %)	113 (97.4%)	144	50.751	0.0001
	> 130 mmHg	26 (45.6%)	3 (2.6%)	29		
Diastolic BP	< 85 mmHg	41 (71.9 %)	114 (98.2%)	155	27.645	0.0001
	> 85 mmHg	16 (28.1%)	2 (1.8%)	18		
Fasting blood sugar	< 100 mg/dl	39 (68.4%)	114 (98.2%)	153	28.961	0.0001
	> 100 mg/dl	18 (31.6%)	2 (1.8%)	20		
High-density lipoprotein	< 40 mg/dl	13 (22.8%)	3 (2.6%)	16	18.617	0.001
	> 40 mg/dl	44 (77.2%)	113 (97.4%)	157		
Triglycerides	< 150 mg/dl	48 (84.2%)	114 (98.2%)	162	11.974	0.01
	> 150 mg/dl	9 (15.8%)	2 (1.8%)	11		

Table 5 shows the association between MetS and sociodemographic factors among the children. Significant associations were evident between MetS and age, gender, income status, and type of family, with a p-value <0.05.

**Table 5:** Association between metabolic syndrome and sociodemographic variables APL: above poverty line; BPL: below poverty line * Indicates significance of p-value at <0.05 level

Variable		Group		Total	X^2^	p-value
		Case (n = 57)	Control (n = 116)			
Age in years	11.0–12.0	9 (15.7 %)	35 (30.0%)	44	4.169	0.041*
	>12.0 –13.0	48 (84.3%)	81 (70.0%)	129		
Gender	Male	43 (75.4%)	69 (59.4%)	112	4.263	0.039*
	Female	14 (24.6%)	47 (40.6%)	61		
Income	APL	39 (68.4%)	97 (83.6%)	136	5.252	0.022*
	BPL	18 (31.6%)	19 (16.3%)	37		
Type of family	Nuclear	52(91.2%)	84 (72.4%)	136	8.047	0.005*
	Joint/extended	05 (8.8%)	32 (27.5%)	37		
Number of siblings	Sibling	43 (75.4%)	94 (81.0 %)	137	0.726	0.394
	Single child	14 (24.6%)	22 (19.0%)	36		
Birth order	First	40 (70.2%)	83 (71.6%)	123	0.035	0.851
	Second or more	17 (29.8%)	33 (28.4%)	50		

Table 6 shows that a significant association was found between MetS risk and various lifestyle practices such as use of bike/walking to school, organized physical activity (e.g., aerobic exercises, swimming, cycling, dancing) and moderately vigorous physical activity (MVPA) for more than three days in a week, eating three meals in a day, daily consumption of fruits and vegetables, eating food from outside, screen use for more than two hours, sleeping for at least eight hours at night, and preference for different drinks when thirsty (p-value <0.05).

**Table 6:** Association between metabolic syndrome and lifestyle practices PE: physical education; MVPA: moderately vigorous physical activity * Indicates significance of p-value at <0.05 level

Variable		Group		Total	X^2^	p-value
		Cases (n = 57), n (%)	Controls (n = 116), n (%)			
Use of bike/walking to school	Yes	07 (12.3%)	37 (31.9%)	44	12.287	0.002*
	No	50 (87.7%)	79 (69.1%)	129		
Participation in PE	Yes	54 (94.7%)	104 (89.7%)	158	0.666	0.610
	No	03 (5.3%)	12 (10.3%)	15		
Organized physical activity	Yes	12 (21.0%)	77 (66.4%)	89	31.435	0.0001*
	No	45 (79.0%)	39 (33.6 %)	84		
MVPA or play for 60 minutes a day in a week	Less than 3 days	45 (79.0%)	43 (37.0 %)	88	26.820	0.0001*
	More than 3 days	12 (21.%)	73 (63%)	85		
Eats 3 meals daily	Yes	40 (70.2%)	106 (91.4%)	146	13.046	0.0001*
	No	17 (29.8%)	10 (8.6%)	27		
Eats fruit daily	Yes	25 (43.9%)	70 (60.3%)	95	4.195	0.041*
	No	32 (56.1%)	46 (39.7%)	78		
Eats vegetables daily	Yes	34 (59.6%)	80 (70.0%)	114	6.800	0.009*
	No	23 (40.4%)	36 (30.0%)	59		
Eats junk food	Yes	51 (89.5%)	103 ( 88.8%)	154	0.18	0.893
	No	6 (10.5%)	13 (11.2%)	19		
Eats food from outside twice or more a week	Yes	51 (89.5%)	103 (88.8%)	154	7.092	0.008*
	No	6 (10.5%)	13 (11.2%)	19		
Prefers to drink water when thirsty	Yes	42 (73.7%)	104 (89.7%)	146	7.402	0.005*
	No	15 (26.3%)	12 (10.3%)	27		
Eats with TV or any other screen on	Yes	45 (78.9%)	65 (56%)	110	8.666	0.003*
	No	12 (21.0%)	51 (44%)	63		
Screen time in a day	More than 2 hours	49(86.0%)	80 (69.0%)	129	6.265	0.012*
	Less than 2 hours	08 (14.0%)	36 (31.0%)	44		
Sleeps for at least 8 hours at night	Yes	53(92.9%)	89 (76.7%)	142	6.869	0.009*
	No	04 (7.1%)	27 (23.3%)	31		

Multivariate analysis

Multivariate analysis was carried out on two criteria: variables with p-values <0.25 in univariate analysis and variables with literature evidence of biological plausibility as an associated factor.

Table 7 shows the details of variables that were identified as associated factors after the multivariate analysis. The independent variables that retained their significance (p-value <0.05) after multivariate analysis were gender, organized physical activity, use of bike/walking to school, MVPA or play involving exertion with increased heart rate or respiratory rate, skipping of meals, preference for drinks other than water when thirsty, screen time, eating with screens on, and eight hours of sleep at night. Boys were found to be at 3.8 times more risk of developing MetS than girls (OR: 3.842, 95%CI: 1.227-12.025). The factors identified also included reduced physical activity, especially no organized physical activity (OR: 12.314, 95%CI: 4.655-32.573). The children who performed MVPA or play (OR: 4.436, 95%CI: 1.611-12.220) involving exertion with increased heart or respiratory rates were less likely to have MetS. The children who traveled in vehicles to school were 5.3 times more likely to develop MetS (OR: 5.323, 95%CI: 1.616-17.543) than those who used bikes or walked to school. The children who skipped meals were 5.19 times more likely to develop MetS than those who ate three main meals daily (OR: 5.195, 95%CI: 1.038-25.985). Those who preferred drinks other than water (soft drinks and carbonated drinks) while thirsty were more likely to develop MetS than those who preferred water (OR: 6.535, 95%CI: 1.904-22.429). Screen time of more than two hours daily was an independent variable associated with a 3.1 times greater risk of MetS (OR: 3.102, 95%CI: 1.005-9.57). The children who slept less than eight hours at night were 5.4 times more prone to MetS (OR: 5.446, 95%CI: 1.198-24.746).

**Table 7:** Factors associated with metabolic syndrome using multivariate analysis MVPA: moderately vigorous physical activity; BPL: below poverty line * Indicates significance of p-value at <0.05 level The Goodness of Fit of the model was assessed by Hosmer–Lemeshow Test. The model fit the data well as our logistic regression model showed a non-significant Chi square (Chi-square 4.109 at df 8, Significance 0.847)

Variable	Crude OR	Adjusted OR	95% CI		p-value
			Lower	Upper	
Gender (Reference-Female Gender)	3.895	3.842	1.227	12.025	0.021*
Age (Reference -12-13 years)	1.162	0.337	0.096	1.189	0.091
Income (Reference -BPL)	2.356	0.442	0.111	1.756	0.246
Type of family (Reference - joint or extended family)	3.962	1.070	0.186	6.147	0.940
Use of bike/walking to school (Reference -Yes)	3.345	5.323	1.616	17.534	0.006*
Organized physical activity (Reference -Yes)	5.118	12.314	4.655	32.573	0.000*
More than 3 days of MVPA or play for 60 minutes in a week (Reference -Yes)	9.743	4.436	1.611	12.220	0.004*
Eats 3 meals daily (Reference -Yes)	2.671	5.195	1.038	25.985	0.044*
Eats fruit daily (Reference -Yes)	1.086	0.800	0.306	2.088	0.648
Eats vegetables daily (Reference -Yes)	1.239	1.141	0.394	3.302	0.807
Eats junk food twice or more a week (Reference -No)	2.564	1.162	0.402	3.357	0.782
Prefers to drink water when thirsty (Reference -Yes)	3.095	6.535	1.904	22.429	0.002*
Eats with TV or any other screen on (Reference -No)	2.942	4.212	1.406	12.851	0.010*
Screen time of more than 2 hours a day (Reference -Less than 2 hours)	2.756	3.102	1.005	9.57	0.049*
Sleeps for at least 8 hours at night (Reference -Yes)	6.321	5.446	1.198	24.746	0.028*

Predictive power of associated factors

ROC analysis evaluated the predictive power of the factors associated with pediatric MetS. Table 8 shows an area under the curve (AUC) of 0.926 (95% CI: 0.891-0.961, p < 0.001), indicating high predictive power. The optimal cutoff point for the predictor variables was determined to be 0.5. Our findings suggest that the factors identified in multivariate analysis strongly predict MetS in children. Only those significant variables in the multivariable logistic regression were selected to create the final model which included the male gender, not using the bike or not walking to school, not participating in organized physical activity, not performing MVPA, or not playing for three or more days a week, not taking three meals a day, not prefer to drink water while thirsty, screen time for more than two hours daily, and night sleep for less than eight hours.

**Table 8:** Area under the curve ^a ^Under the nonparametric assumption; ^b^ Null hypothesis: true area = 0.5 *Predicted probability has at least one tie between the positive actual state group and the negative actual state group. Statistics may be biased.

Test result variable(s)*: Predicted probability				
Area	Std. Error^a^	Asymptotic Sig.^b^	Asymptotic 95% Confidence Interval	
			Lower Bound	Upper Bound
.931	.020	.000	.892	.970

**Figure 3:**
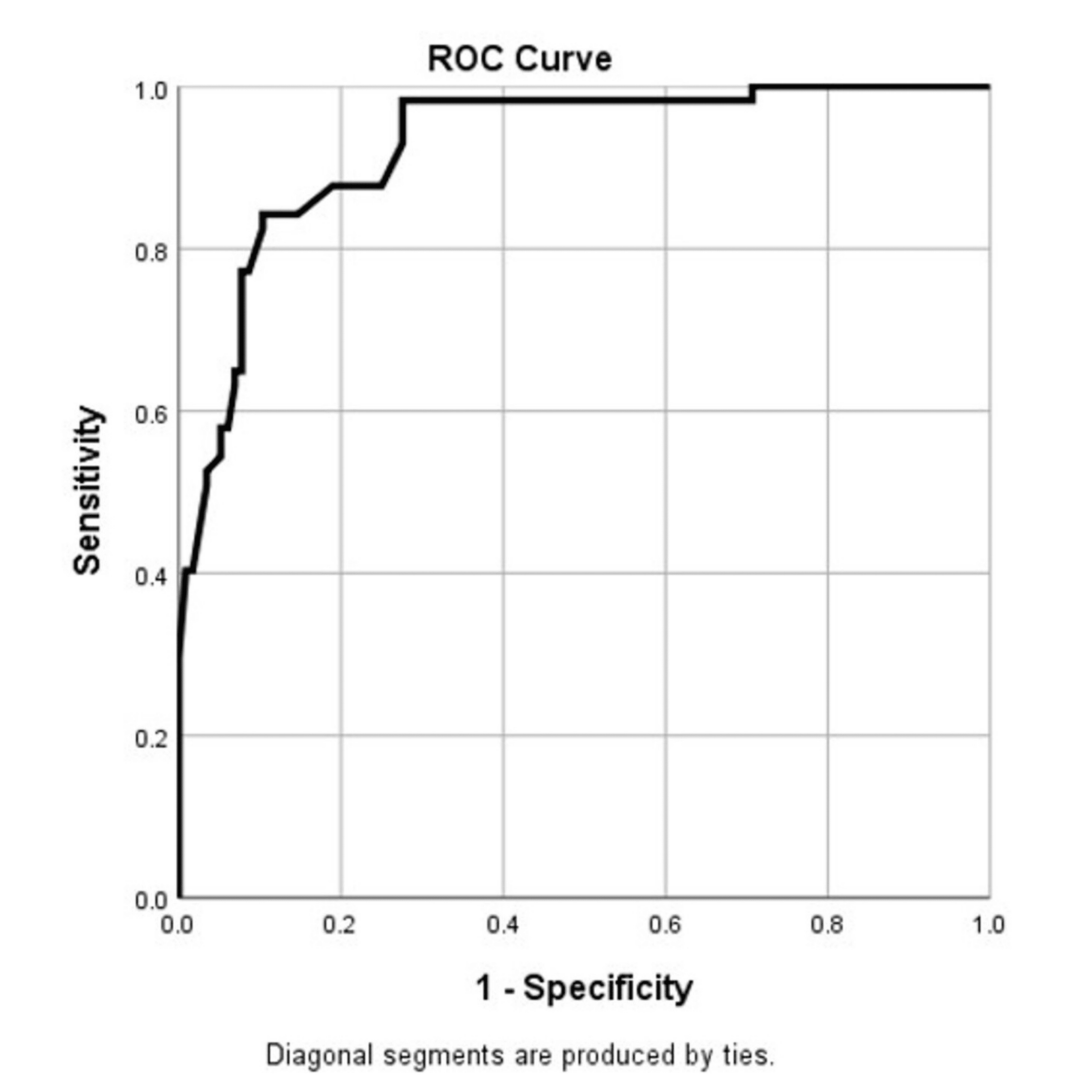
Receiver operating characteristic (ROC) graph

The ROC graph indicates that the model is good at explaining the presence of risk factors and disease occurrence (Figure 3). AUC of 0.931 indicates more than 90% accuracy in identifying risk factors and occurrence of disease. Only those significant variables in the multivariable logistic regression were selected to create the final model which included the male gender, not using the bike or not walking to school, not participating in organized physical activity, not performing MVPA, or not playing for three or more days a week, not taking three meals a day, not preferring to drink water while thirsty, screen time for more than two hours daily, and night sleep for less than eight hours.

Table 9 shows the model summary, indicating the better fit of the logistic regression model. The model suggests good convergence and stability as the estimation terminated after a few iterations.

**Table 9:** Model summary ^a ^Estimation terminated at iteration number 6 because parameter estimates changed by less than .001.

−2 Log likelihood	Cox & Snell R-squared	Nagelkerke R-squared
122.494^a^	0.429	0.596

## Discussion

The current study aimed to find the prevalence of MetS and its associated risk factors among schoolchildren aged 11-13 years. The prevalence of MetS in this study was estimated to be 3.6% among schoolchildren aged 11-13 years. This is in line with the population-based study conducted in different states of India, which projected a prevalence range of less than 1% in Punjab to 17% in Manipur [9]. The global prevalence of MetS among children and adolescents in 2020 is estimated to be at 2·8% for children and 4·8% for adolescents. In children, the prevalence of MetS was 2·2% in high-income countries, 3·1% in upper-middle-income countries, 2·6% in lower-middle-income countries, and 3·5% in low-income countries [17].

The multivariate analysis showed that boys have a 3.8 times higher risk of developing MetS than girls (OR: 3.842, 95%CI: 1.227-12.025). This is congruent with a school‑based cross‑sectional study conducted during 2015-2016 in Himachal Pradesh, India, which found that male participants were at a 2.3 times higher risk of developing pediatric MetS than female participants [10]. The factors found to be associated with MetS in this study include physical activity, especially organized physical activity (OR: 12.314, 95%CI: 4.655-32.573). Children who took part in MVPA or play (OR: 4.436, 95%CI: 1.611-12.220) involving exertion with increased heart rate or respiratory rate were found to have less chance for MetS. Children who travelled in vehicles to school were 5.3 times more likely to develop MetS (OR: 5.323, 95%CI: 1.616-17.543) than those who used bikes or walked to school. These findings are consistent with the findings of school-based research conducted in October 2016 in Addis Ababa, which established that adolescents who did not perform vigorous or moderate physical activity were 1.56 times more at risk of developing MetS than adolescents who performed vigorous or moderate physical activity [18]. Replacement of prolonged sedentary time with MVPA may be the preferred option for behaviour change, given the beneficial associations with a wide range of cardiometabolic risk factors (including adiposity, HDL cholesterol, high BP, and clustered cardiometabolic risk [19].

It was also found that spending more than two hours at a screen daily was an independent variable associated with a 3.1 times higher risk of MetS (OR: 3.102, 95%CI: 1.005-9.57). These findings were supported by those from previous studies of sedentary lifestyle which showed that sedentary activity (adjusted OR: 3.57, 95%CI: 1.48-8.59) was a significant factor associated with MetS in children with obesity [12]. Using a multivariate model, a multicentric cross-sectional study of 4,200 school students aged 7-18 years in 30 provinces of Iran found that individuals who slept less than eight hours a day had significantly higher odds of MetS (OR: 2.05, 95%CI: 1.19-3.63) [20], which supports our findings that showed that children who slept less than eight hours at night were 5.4 times more times prone to MetS (OR: 5.446, 95%CI: 1.198-24.746) than those who slept more than eight hours.

The perpetual rise of pediatric obesity worldwide has increased the likelihood of associated sequelae. In MetS, because of excess fat content, dysfunction of its core visceral adipocytes occurs and hence the occurrence of any risk factor may result in cardiovascular or metabolic diseases like type 2 diabetes [21]. MetS among children and adolescents is a growing public health problem in low- and middle-income countries, where the prevalence of obesity is on the drift. Preventive interventions such as community and school-based activities need to be outlined. Elaborating physical activities and healthy eating could forestall this issue [22].

Limitations

This study has several limitations; however, we could establish strong predictors and risk factors of MetS among children. The limitations of this study were that the questionnaire and lifestyle checklist were used for the first time. Moreover, the lifestyle checklist did not include the exact duration of physical activity. A pediatric lifestyle checklist should have been developed for the study. The study objective was limited to children in the age group of 11-13 years. Genetic and other nonmodifiable risk factors were not included in the study's perspective.

## Conclusions

This nested, case-control study identified key risk factors for MetS among schoolchildren. These factors include modifiable ones such as physical activity, especially organized physical activity and MVPA or play, skipping meals, reduced sleep at night, eating with screens on, use of screens for more than two hours daily, preference for soft drinks or carbonated drinks over water when thirsty, and sleeping for less than eight hours during night. Our findings support the need for early intervention to prevent MetS and its long-term consequences among children. The findings also imply the need for school-based physical activities to reduce sedentary behavior. We foresee our findings shall contribute to the development of targeted interventions and strategies to decrease the affliction of MetS in children and bolster healthy lifestyles from an early age.

## Figures and Tables

**Figure 4:**
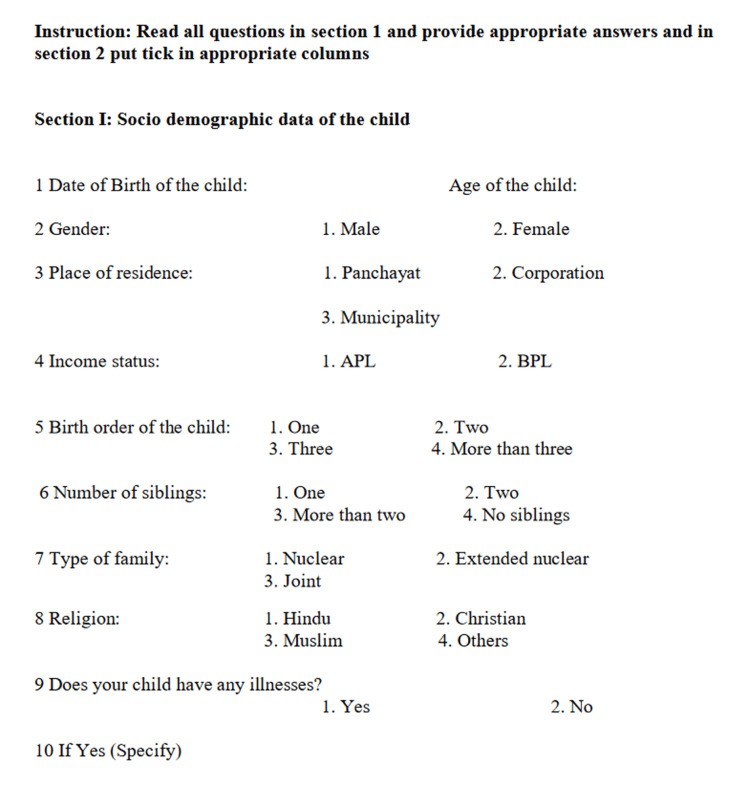
Sociodemographic Questionnaire

**Figure 5:**
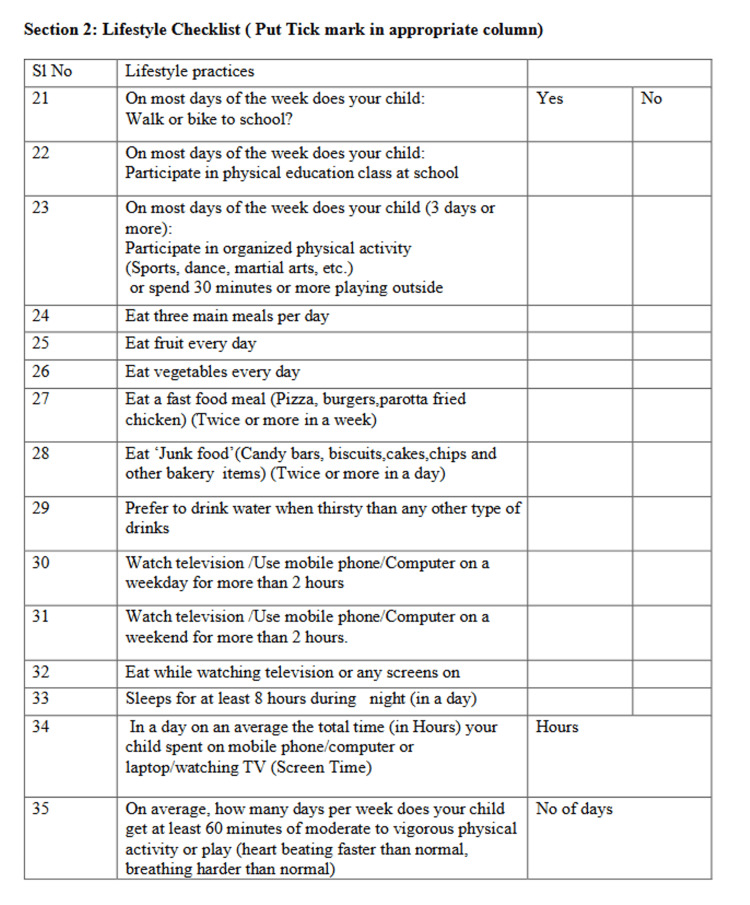
Lifestyle Checklist

## Appendices

 Parental questionnaire and lifestyle checklist used in the study
